# Electrical storm: Incidence, Prognosis and Therapy

**Published:** 2011-03-25

**Authors:** Riccardo Proietti, Antonio Sagone

**Affiliations:** Cardiac Electrophysiology Laboratory, Luigi Sacco Hospital Milano, Italy

**Keywords:** Electrical storm, incessant arrhythmias, radiofrequency transcathether ablation

## Abstract

Implantable defibrillators are lifesavers and have improved mortality rates in patients at risk of sudden death, both in primary and secondary prevention.  However, they are unable to modify the myocardial substrate, which remains susceptible to life-threatening ventricular arrhythmias. Electrical storm is a clinical entity characterized the recurrence of hemodynamically unstable ventricular tachycardia and/or ventricular fibrillation, twice or more in 24 hours, requiring electrical cardioversion or defibrillation. With the arrival of the implantable cardioverter-defibrillator, this definition was broadened, and electrical storm is now defined as the occurrence of three or more distinct episodes of ventricular tachycardia or ventricular fibrillation in 24 hours, requiring the intervention of the defibrillator  (anti-tachycardia pacing or shock). Clinical presentation can be very dramatic, with multiple defibrillator shocks and hemodynamic instability. Managing its acute presentation is a challenge, and mortality is high both in the acute phase and in the long term. In large clinical trials involving patients implanted with a defibrillator both for primary and secondary prevention, electrical storm appears to be a harbinger of cardiac death, with notably high mortality soon after the event. In most cases, the storm can be interrupted by medical therapy, though transcatheter radiofrequency ablation of ventricular arrhythmias may be an effective treatment for refractory cases.

This narrative literature review outlines the main clinical characteristics of electrical storm and emphasises critical points in approaching and managing this peculiar clinical entity. Finally focus is given to studies that consider transcatheter ablation therapy in cases refractory to medical treatment.

The term electrical storm (ES) was introduced in the 1990s to describe a state of electrical instability of the heart characterized by a series of malignant ventricular arrhythmias in a short period of time [1]. This condition has been described in patients with post-infarction ischemic heart disease, various forms of cardiomyopathy, valve disease, corrected congenital heart disease and genetically determined heart diseases with no apparent structural alteration, as for example in Brugada syndrome [2]. The rapid succession of life-threatening ventricular arrhythmias leads to increased mortality and requires intensive care, hemodynamic study, multiple cardioversions and cardiopulmonary resuscitation [2].

ES was formerly defined as the recurrence of hemodynamically unstable ventricular tachycardia and/or ventricular fibrillation, twice or more in 24 hours, requiring electrical cardioversion or defibrillation [3-6]. With the arrival of the ICD (implantable cardioverter-defibrillator) this definition was broadened, and ES is now defined as the occurrence of three or more distinct episodes of ventricular tachycardia (VT) or ventricular fibrillation (VF) in 24 hours, requiring the intervention of the defibrillator  (anti-tachycardia pacing [ATP] or shock) [7-21].  It should be noted that this latter definition does not include the presence of hemodynamic instability, since the intervention of the defibrillator generally prevents the arrhythmia from becoming hemodynamically significant. Obviously, an inappropriate intervention of the device is not considered [10]. Moreover, the episodes of VT must be separate, meaning that the persistence of ventricular tachycardia following inefficacious intervention is not regarded as a second episode [10]. By contrast, a sustained ventricular tachycardia that resumes immediately after (≥1 sinus cycle and within 5 minutes) efficacious therapeutic intervention by the defibrillator is regarded as a severe form of electrical storm [10]. The electrical instability that may arise in the first week following ICD implantation is thought to be caused by an irritative stimulus and is not strictly interpreted as an electrical storm [10].  The inclusion of anti-tachycardia pacing in the defining criteria of ES requires particular attention for two reasons: first, the fact that it does not arouse immediate alarm and may cause the real incidence of the phenomenon to be underestimated; secondly, a case of a single shock by the defibrillator requires careful evaluation by the cardiologist, since it might in reality, conceal an ES in which other tachyarrhythmias have been treated by means of anti-tachycardia pacing.  ES is deemed to be resolved if the patient is free from VT for at least two weeks [14].

## Incidence

Between 1996 and 2006, several studies were carried out in order to investigate the incidence and prognosis of ES in ICD recipients (Table 1). However, the definition of ES was not homogeneous in the studies examined. This fact, together with the differences in the considered observation period and in the populations assessed, yielded great variability in the incidence of ES reported in the various studies. According to the above-mentioned definition (3 defibrillator interventions in 24 hours), the reported incidence of ES varies from 10 to 28% in an observation period of 1-3 years in those studies in which ICD implantation was carried out for secondary prevention [9,11,15]. In the MADIT II study, which concerned primary prevention, the incidence proved to be substantially lower- about 4% [12].

The time from ICD implantation to the onset of electrical storm also differs in the various case-records. Indeed, the time-lapse seems to be particularly affected by the population considered, the myocardial substrate, the medical therapy undertaken and the indications for device implantation.  In one of the earliest studies [9], the average time of ES onset was reported to be 4-5 months after device implantation. By contrast, more recent studies have reported a period of 2-3 years [16,18].

The number of VT that occurs during an episode of electrical storm varies substantially among the different studies. Verma reported an average of 5±5 shocks [18], while Green [14] recorded an average of 55 VT.  Most of the arrhythmic episodes that occur during ES are episodes of monomorphic ventricular tachycardia, while polymorphic VT and VF are unusual causes [10]. Verma [18] reports a significant correlation between the initial arrhythmia that prompted ICD implantation and the arrhythmia responsible for ES.

## Triggers and risk factors

Many papers report that in the majority of patients, an evident trigger i.e. a contingent cause that prompts the storm, cannot be identified [10]. Credner [9] underlined the presence of hypokalemia, acute coronary syndrome and worsening heart failure as potential triggers in 26% of the patients in his case-records. Similarly, the SHIELD trial [17], which evaluated the effect of azimilide on the frequency of defibrillator shock, identified a storm trigger in 13% of patients: worsening heart failure in 9% and electrolytic imbalance in 4%. By contrast, the papers by Green [14] and Bansch [15] respectively report an identifiable trigger in 71% and 65% of their patients. In both studies, psychological stress seemed to be a trigger, defining 10% of the causes detected by Green [14] and 4% of those reported by Bansch [15]. This finding seems to be determined by adrenergic activation, the impact of which on the genesis of the storm is corroborated by the finding of an increased incidence during the daytime hours, a marked effect of beta-blocker therapy and a reduced sensitivity of baroreceptor reflexes.

Like triggers, the risk factors for ES are also difficult to identify. Severely compromised ventricular function, chronic renal insufficiency and ventricular tachycardia as the onset arrhythmia all seem to correlate significantly with the development of storms [19].  In the MADIT II study [12], patients with acute coronary syndrome or episodes of tachycardia and/or ventricular fibrillation after enrolling showed a higher incidence of ES.

Although these data on the causes and risk factors are not conclusive, it emerges that ES is the result of multiple interactions between a predisposing electrophysiological substrate and alterations in the autonomous nervous system and cellular milieu [10]. The progression of myocardial disease through fibrosis, ischemia and ventricular remodeling may manifest itself as an isolated tachyarrhythmic episode that is predictive of future ES. The correlation among worsening heart disease, acute disease and emotional stress corroborates the critical role of an increased activation of the sympathetic nervous system in the pathogenesis of ES [10].

## Prognosis

The immediate clinical consequence of ES is hospitalization, which takes place in 80% of patients, particularly those who have received a shock from the device (100% if >3 shocks are received) [15]. Moreover, the electrical instability impairs the patient's quality of life and can induce a state of anxiety, which may have psychological repercussions [10].

With regard to mortality, it is not surprising that studies conducted on large numbers of patients for a sufficiently long period of follow-up have documented an increase.  In the AVID study [11], patients with ES displayed a 2-fold higher risk of all-cause mortality; this risk was particularly concentrated in the first 3 months following the event. On considering patients with non-ischemic heart disease, Bansch [15] found a higher mortality rate over a follow-up of 3 years in patients with a previous episode of ES; this risk was even greater if the electrical instability had caused acute heart failure. The MADITI II study [12] also reported a higher mortality rate among patients with ES, again concentrated in the first 3 months after the event.

Verma [18] also observed that mortality was higher among patients with ES than among control subjects with ICD.  However, this increased mortality occurred much later than the first 3 months reported in the previous studies and proved to correlate with the progression of heart failure rather than arrhythmic death. This finding raises the question of how ES contributes to worsening the prognosis of such patients. Undoubtedly, it can be hypothesized that the shocks exert an effect on the myocardial damage, inflammation and even remodeling; however, any such effect should not be overestimated [21].  Indeed, episodes of ventricular fibrillation can lead to an increase in intracellular calcium and the progression of heart failure [21].

Such mechanisms remain plausible, though unconfirmed. What is clear, however, is that, while immediate mortality linked to ES in ICD patients is not high, the first few months after the episode seem to be critical on account of the increased mortality that is related not so much to the potential arrhythmic instability as to the progression of cardiac dysfunction [21]. This finding suggests that ES is a critical moment in the progression towards irreversible heart failure, of which it may also constitute the initial manifestation [14].

## Pharmacological therapy

Electrical storm constitutes a critical clinical situation both on account of the difficulty in approaching and managing hemodynamically unstable ventricular arrhythmias and because it is associated with significant pain and anxiety, which further increase the sympathetic tone and facilitate further arrhythmias [20].

A patient with ES has to be hospitalized and monitored in an intensive care unit. The most urgent evaluation concerns the hemodynamic stability of the arrhythmias and, if they degenerate into acute heart failure, prompt assessment of the complications linked to this (such as pulmonary enema or acute renal insufficiency).

When a trigger can be identified, its correction may reverse the electrical instability of the myocardium.  For this reason, thorough clinical and laboratory evaluation is of fundamental importance in order to search for possible pro-arrhythmic triggers, such as hydro-electrolytic imbalance or the intensification of myocardial ischemia [20]. If any of these triggers is detected, it must be promptly treated. In some cases, myocardial revascularization is necessary; equally often, however, electrical stabilization is required [20]. The intravenous administration of magnesium and potassium may be undertaken in patients with QT lengthening or hypokalemia [20]. With regard to immediate drug therapy, a beneficial effect can be achieved by blocking the sympathetic system through the intravenous administration of beta-blockers combined with sedatives, such as benzodiazepine [22].

In the absence of contraindications (such as QT lengthening or polymorphic ventricular tachycardia), amiodarone is generally the antiarrhythmic drug of choice and has been validated in numerous clinical trials [3,5]. If the intravenous combination of amiodarone and beta-blockers proves inefficacious, the addition of lidocaine is a reasonable option [23].

For what concerns the prevention of ES, interesting results have been yielded by some drugs such as azamilide, a class III antiarrhythmic which blocks the calcium channels and prolongs the refractory period [21].

Nevertheless, the principal factor in the prevention of electrical instability is correct ICD programming [21]. Given that sympathetic hyperactivation is an important trigger, the risk of shock would be minimized, all the more so as anti-tachycardia pacing can successfully terminate a significant percentage of ventricular tachycardias [21]. In some cases, greater electrical stabilization can be achieved by shifting to cardiac resynchronization therapy (CRT) by means of biventricular pacing (which necessitates replacement of the ICD and implantation of a lead in the coronary sinus for left ventricular pacing).  This strategy is supported by a very recent study by Nordbeck [24], in which the incidence of ES was seen to be much lower in CRT patients than in ICD patients undergoing pacing in the left ventricle alone. In addition, a sub-analysis of the Italian Insync ICD registry [25] has revealed that the incidence of ES is lower in patients on CRT and that those who do not respond to CRT are at greater risk.

## Transcatheter radiofrequency ablation therapy

In cases that are refractory to drug therapy, transcatheter radiofrequency ablation of the arrhythmogenic myocardial substrate can be carried out during ES.  This was first described in a few case reports [26-29] and subsequently presented in ample case-records (Table 2).

Willems et al. [30] were the first to utilize transcatheter radiofrequency delivery in a series of 6 patients with sustained ventricular tachycardia; they identified the site of emergence of the arrhythmia through early activation on mapping during tachycardia and through pace-mapping.  In their case-records, acute success was 100%; in all cases, the arrhythmia was interrupted and, at the end of the procedure, could no longer be induced.  No major procedural complications were recorded.  One patient died of acute heart failure 24 hours after the ablation procedure. However, the rate of recurrence of single episodes of ventricular arrhythmia was 80%.

Strickbeger [31] reported that radiofrequency ablation significantly reduced ICD interventions in a series of 21 patients who had received frequent defibrillator shocks for sustained  ventricular arrhythmias refractory to medical therapy.

Sra [32] was the first to describe the advantage of electroanatomical mapping in the ablation of ES in a series of 19 patients; no recurrence of arrhythmia was recorded in 66% of cases over a 26-week follow-up.  In that study, only one case of cardiac tamponade occurred during ablation, and this was successfully drained.  No deaths or long-term complications were recorded during the follow-up period considered.  Similarly efficacious results were described by Schreieck [33] in 5 patients with ischemic heart disease who underwent transcatheter ablation (during follow-up of 12-30 months, 3 were free from arrhythmia recurrence, while 2 suffered single episodes of VT).  No intra- or periprocedural complications arose.

Silva [34] reported the efficacy of transcatheter ablation in a group of 15 patients with ES who were heterogeneous in terms of underlying heart disease (ischemic in 9 cases, idiopathic in 4, arrhythmogenic dysplasia of the right ventricle in 1, and no structural heart disease in 1).  Ablation of the clinical tachycardia was achieved in 80% of cases. No intra- or periprocedural complications were recorded and, over a follow-up of 12±17 months, only two patients suffered a single recurrence of VT.

Marrouche [35] reported on 29 patients with ischemic heart disease in whom recurrent VF was triggered by monomorphic ventricular extrasystole originating in the fibrous peri-infarction zone. In 8 patients in whom ES was refractory to drug therapy, ablation of the ventricular extrasystole was successfully performed after mapping, and control of ES was achieved. Over a follow-up period of more than 1 year, a single episode of VF occurred in one patient and an episode of monomorphic VT occurred in another.

Bansch [36] reports the case-records of 4 patients with ES following acute myocardial infarction in whom control of the ventricular tachyarrhythmias was achieved through ablation of the premature ventricular beats underlying the arrhythmia.

In 10 patients with sustained ventricular arrhythmia, Brugada [37] reported that epicardial ablation was able to control ES in 8 cases. No procedural complications were recorded and no deaths occurred during follow-up (18±18 months).

Carbucicchio [38] reports the experience of transcatheter ablation as the emergency therapy of choice in a large heterogeneous population of patients with ES refractory to drug therapy in an important Italian cardiology center. In 95 patients suffering from idiopathic ischemic heart disease and arrhythmogenic dysplasia of the right ventricle, radiofrequency transcatheter ablation was able to suppress ES in the acute phase in all cases and achieved non-inducibility of ventricular arrhythmias at the end of the procedure in 89%.  On 2-year follow-up examination, 92% of patients were free from ES recurrence and 66% were free from VT recurrence.  No major procedure-related complications were recorded.

## Conclusions

Electrical storm is a very challenging clinical event that can be considered under two perspectives: the arrhythmic event can constitute the clinical manifestation of worsening heart failure or it might compromise myocardial function, thereby giving rise to the high long-term mortality seen in these patients.

Treatment in the acute phase is off-label and often requires the simultaneous intravenous administration of several antiarrhythmic drugs.

Recent studies have proposed transcatheter ablation in cases of electrical storm that are refractory to drug therapy. In several case-records, this approach proved to be safe and effective in interrupting the rapid sequence of life-threatening ventricular arrhythmias, nevertheless it does not seem to change the long-term prognosis of these patients, who continue to bear an increased burden of mortality.

## Figures and Tables

**Table 1:**
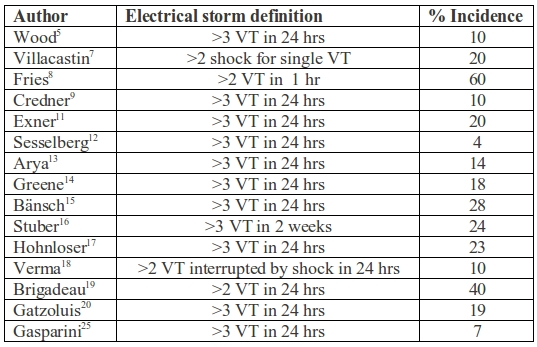
Incidence of electrical storm.

VT=ventricular tachycardia, hrs=hours

**Table 2:**
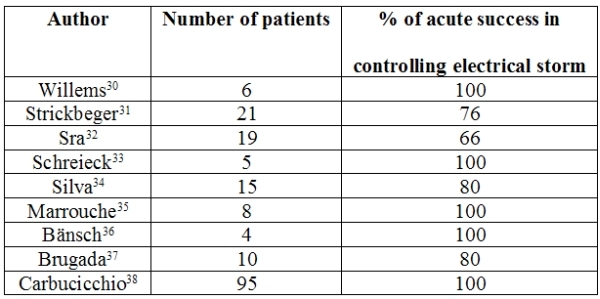
Success rate of transcathether radiofrequency abation of electrical storm.

## References

[R1] Kowey PR (1996). An overview of anti-arrhythmic drug management of electrical storm. Can J Cardiol.

[R2] Bedell SE (1983). Survival after cardiopulmonary resuscitation in the hospital. N Engl J Med.

[R3] Kowey PR (1995). Randomized, double-blind comparison of intravenous amiodarone and bretylium in the treatment of patients with recurrent, hemodynamically destabilizing ventricular tachycardia or fibrillation. The Intravenous Amiodarone Multicenter Investigators Group. Circulation.

[R4] Dorian P (1997). An overview of the management of electrical storm. Can J Cardiol.

[R5] Wood MA (1995). Long term temporal patterns of ventricular tachyarrhythmias. Circulation.

[R6] Scheinman MM (1995). Dose ranging study of intravenous amiodarone in patients with life-threatening ventricular tachyarrhythmias. Circulation.

[R7] Villacastin J (1996). Incidence and clinical significance of multiple consecutive, appropriate, high-energy discharges in patients with implanted cardioverter defibrillator. Circulation.

[R8] Fries R (1997). Incidence and clinical significance of short term recurrent ventricular tachyarrhythias in patients with implantable cardeioverter-defibrillator. Int J Cardiol.

[R9] Credner SC (1998). Electrical storm in patients with transvenous implantable cardioverter-defibrillators: incidence, management and prognostic implications. J Am Coll Cardiol.

[R10] Israel CW (2007). Electrical storm in patients with an implanted defibrillator: a matter of definition. Ann Noninvasive Electrocardiol.

[R11] Exner DV (2001). Electrical storm presages nonsudden death: the Antiarrhythmics Versus Implantable Defibrillators (AVID) trial. Circulation.

[R12] Sesselberg HW (2007). Ventricular arrhythmia storms in postinfarction patients with implantable defibrillators for primary prevention indications: a MADIT-II substudy. Heart Rhythm.

[R13] Arya A (2006). Prevalence and predictors of electrical storm in patients with implantable cardioverter defibrillators. Am J Cardiol.

[R14] Greene M (2000). Is electrical storm in ICD patients the sign of a dying heart? Outcome of patients with clusters of ventricular tachyarrhythmias. Europace.

[R15] Bansch D (2000). Clusters of ventricular tachycardias signify impaired survival in patients with idiopathic dilated cardiomyopathy and implantable cardioverter defibrillators. J Am Coll Cardiol.

[R16] Stuber T (2005). Characteristics and relevance of clustering ventricular arrhythmias in defibrillator recipients. Pacing Clin Electrophysiol.

[R17] Hohnloser SH (2006). Electrical storm in patients with an implantable defibrillator: incidence, features, and preventive therapy: Insights from a randomized trial. Eur Heart J.

[R18] Verma A (2004). Prevalence, predictors, and mortality significance of the causative arrhythmia in patients with electrical storm. J Cardiovasc Electrophysiol.

[R19] Brigadeau F (2006). Clinical predictors and prognostic significance of electrical storm in patients with implantable cardioverter defibrillators. Eur Heart J.

[R20] Gatzoulis KA (2008). Electrical storm: a new challenge in the age of implantable defibrillators. Hellenic J Cardiol.

[R21] Huang DT (2008). Recurrent ventricular arrhythmia storms in the age of implantable cardioverter defibrillator therapy: a comprehensive review. Progress cardiovasc Diseases.

[R22] Nademanee K (2000). Treating electrical storm: Sympathetic blockade versus advanced cardiac life support-guided therapy. Circulation.

[R23] Fuchs T (2008). Use of a combination of class III and class Ic anti-arrhythmic agents in patients with electrical storm. Phamacotherapy.

[R24] Nordbeck P (2010). Effects of cardiac resynchronization therapy on the incidence of electrical storm. Int J Cardiol.

[R25] Gasparini M (2008). Electrical storm in patients with biventricular implantable cardioverter defibrillator: incidence, predictors and prognostic implications. Am Heart J.

[R26] Kolettis TM (2005). Radiofrequency catheter ablation for electrical storm in a patient with dilated cardiomyopathy. Hell J Cardiol.

[R27] Nakagawa E (2008). Successful radiofrequency catheter ablation for electrical storm of ventricular fibrillation in a patient with Brugada Syndrome. Circ J.

[R28] Enjoji Y (2006). Catheter ablation for an incessant form of antiarrhythmic drug-resistant ventricular fibrillation after acute coronary syndrome. PACE.

[R29] Thoppil PS (2008). Successful catheter ablation of persistent electrical storm late post myocardial infarction by targeting Purkinje arborization triggers. Indian Pacing Electrophysiol J.

[R30] Willems S (1993). Radiofrequency catheter ablation of ventricular tachycardia following implantation of an automatic cardioverter defibrillator. PACE.

[R31] Strickberger AS (1997). A prospective evaluation of catheter ablation of ventricular tachycardia as adjuvant therapy in patients with coronary heart disease and an implantable cardioverter defibrillator. Circulation.

[R32] Sra J (2001). Electroanatomically guided catheter ablation of ventricular tachycardias causing multiple defibrillator shocks. PACE.

[R33] Schreieck J (2005). Rescue ablation of electrical storm in patients with ischemic cardiomyopathy: a potential-guided ablation approach by modifying substrate of intractable, unmappable ventricular tachycardias. Heart Rhythm.

[R34] Silva R (2004). Radiofrequency Catheter Ablation for Arrhythmic Storm in Patients with An Implantable Cardioverter Defibrillator. PACE.

[R35] Marrouche NF (2004). Mode of initiation and ablation of ventricular fibrillation storms in patients with ischemic cardiomyopathy. J Am Coll Cardiol.

[R36] Bansch D (2003). Successful catheter ablation of electrical storm after myocardial infarction. Circulation.

[R37] Brugada J (2003). Nonsurgical transthoracic epicardial radiofrequency ablation. An alternative in incessant ventricular tachycardia. J Am Coll Cardiol.

[R38] Carbucicchio C (2008). Catheter ablation for the treatment of electrical storm in patients with implantable cardioverter-defibribrillators. Short and long-term outcomes in a prospective single-center study. Circulation.

